# Advanced Paternal Age in Focus: Unraveling Its Influence on Assisted Reproductive Technology Outcomes

**DOI:** 10.3390/jcm13102731

**Published:** 2024-05-07

**Authors:** Aris Kaltsas, Athanasios Zikopoulos, Dionysios Vrachnis, Chara Skentou, Evangelos N. Symeonidis, Fotios Dimitriadis, Sofoklis Stavros, Michael Chrisofos, Nikolaos Sofikitis, Nikolaos Vrachnis, Athanasios Zachariou

**Affiliations:** 1 Third Department of Urology, Attikon University Hospital, School of Medicine, National and Kapodistrian University of Athens, 12462 Athens, Greece; a.kaltsas@uoi.gr (A.K.); mxchris@yahoo.com (M.C.); 2 Laboratory of Spermatology, Department of Urology, Faculty of Medicine, School of Health Sciences, University of Ioannina, 45110 Ioannina, Greece; nsofikit@uoi.gr; 3 Department of Obstetrics and Gynecology, Royal Cornwall Hospital, Truro TR1 3LJ, UK; athanasios.zikopoulos1@nhs.net; 4 Medical School, National and Kapodistrian University of Athens, 12462 Athens, Greece; dionisisvrachnis@gmail.com; 5 Department of Obstetrics and Gynaecology, Faculty of Medicine, School of Health Sciences, University of Ioannina, 45110 Ioannina, Greece; haraskentou@uoi.gr; 6 Department of Urology, Faculty of Medicine, School of Health Sciences, Aristotle University of Thessaloniki, 54124 Thessaloniki, Greece; evansimeonidis@gmail.com (E.N.S.); difotios@auth.gr (F.D.); 7 Third Department of Obstetrics and Gynecology, Attikon University Hospital, School of Medicine, National and Kapodistrian University of Athens, 12462 Athens, Greece; sfstavrou@med.uoa.gr (S.S.);; 8 Vascular Biology, Molecular and Clinical Sciences Research Institute, St George’s University of London, London SW17 0RE, UK

**Keywords:** advanced paternal age, assisted reproductive technologies, sperm quality, genetic risks, epigenetic shifts, embryo quality, preimplantation genetic testing, delayed fatherhood

## Abstract

As global demographics shift toward increasing paternal age, the realm of assisted reproductive technologies (ARTs), particularly in vitro fertilization (IVF) and intracytoplasmic sperm injection (ICSI), faces new challenges and opportunities. This study provides a comprehensive exploration of the implications of advanced paternal age on ART outcomes. Background research highlights the social, cultural, and economic factors driving men toward later fatherhood, with a focus on the impact of delayed paternity on reproductive outcomes. Methods involve a thorough review of existing literature, centering on changes in testicular function, semen quality, and genetic and epigenetic shifts associated with advancing age. Study results point to intricate associations between the father’s age and ART outcomes, with older age being linked to diminished semen quality, potential genetic risks, and varied impacts on embryo quality, implantation rates, and birth outcomes. The conclusions drawn from the current study suggest that while advanced paternal age presents certain risks and challenges, understanding and mitigating these through strategies such as sperm cryopreservation, lifestyle modifications, and preimplantation genetic testing can optimize ART outcomes. Future research directions are identified to further comprehend the epigenetic mechanisms and long-term effects of the older father on offspring health. This study underscores the need for a comprehensive approach in navigating the intricacies of delayed fatherhood within the context of ART, aiming for the best possible outcomes for couples and their children.

## 1. Introduction

In recent decades, a significant demographic shift has been observed globally, with an increasing number of men choosing to embrace fatherhood later in life [1]. This trend towards deferred paternity, caused and/or influenced by an array of social, cultural, and economic factors, has profound implications for the realm of assisted reproductive technologies (ARTs), particularly in vitro fertilization (IVF) and intracytoplasmic sperm injection (ICSI) [2]. Moreover, the rising prevalence of obesity among reproductive-age men has emerged as a critical factor influencing male fertility, exacerbating issues related to age and reproductive outcomes [3,4]. As couples seek ART services at older ages, the effects of advancing paternal age on reproductive outcomes have become a focal point of intense research and discussion.

This article aims to elucidate the multifaceted landscape of increasing paternal age and its ramifications within the context of ART. It delves into the driving forces behind this demographic shift, such as delayed marriage, extended life expectancies, and the enhanced efficacy of ART [5]. It also critically examines the burgeoning body of research that seeks to unravel the complex biological, psychological, and sociocultural dimensions of this phenomenon. In many countries, there has been a marked increase in the proportion of older fathers involved in live births, reflecting, as mentioned, a broader trend towards delayed childbearing [6]. While the ramifications of increased maternal age on fertility and pregnancy outcomes are well-documented, the implications of advancing paternal age are less clear and present unique challenges [7,8]. It is important to note that the intersection of advancing age and obesity introduces additional complications, such as hormonal changes, oxidative stress, and temperature stress, which are particularly exacerbated by the common occurrence of varicocele in older males, further impairing fertility [9,10,11]. The present work navigates through these complexities, exploring the decline in testicular function and semen quality as well as the potential genetic and epigenetic shifts that accompany advancing paternal age.

This study further investigates the consequential impacts of these changes on ART outcomes, including fertilization rates, embryo quality, implantation success, and the risks of miscarriages and birth complications [12]. It underscores the need for a comprehensive approach that considers both the biological and psychological aspects of delayed fatherhood. The geopolitical, cultural, and psychological dimensions that lead to delayed parenthood are also dissected, elucidating their roles in shaping contemporary paternity trends and the associated mental health considerations [2].

As the landscape of reproductive medicine evolves with these demographic shifts, understanding the intricacies of advanced paternal age becomes crucial. The present paper not only provides a detailed investigation into the current state of knowledge but also discusses strategies for mitigating risks associated with delayed fatherhood and outlines future research directions that can enhance our insight into and management of the effects of advanced paternal age on ART outcomes [13]. The current article comprises a wide-ranging narrative ‘voyage’ covering the cellular, molecular, and anatomical aspects of aging of the male reproductive system all the way to the wider ramifications extending to reproductive health and well-being.

## 2. Trends in Increasing Paternal Age and ART

Increasing paternal age in the context of ART is influenced by a myriad of social, cultural, and economic factors, the increase being variously attributed to delayed marriage, extended life expectancies, and the enhanced efficacy of ART [14]. Recent demographic studies have indicated a noticeable shift in family planning, with both men and women opting to put off parenthood. In England and Wales, the proportion of fathers aged 35–54 years contributing to live births increased from 25% in 1993 to 40% in 2003, illustrating a broader trend towards postponement of childbearing [6]. In the USA, paternal age at the time of childbirth has seen a notable upward trend over the past four decades, with the average age at which men become fathers having risen from 27.4 years in the early 70s to 30.9 in recent times [15].

In numerous nations, guidelines recommend restricting access to ART for men who are younger than 60 years due to the potential impacts on offspring health and ART outcomes. This threshold is based on the general observation of increasing risk with advancing age, although the rationale behind this specific age is not always clearly articulated [16]. However, in order to minimize the potential risks associated with couples using a sperm donation program, various countries have implemented age restrictions for sperm donors. In Great Britain, the age limit is set at 46 years [17], while in the USA it is 40 years [18]. Australia and France have set the age limit at 45 years [19,20], and the European Society of Human Reproduction and Embryology recommends a limit of 50 years [21]. The absence of a true drop in male fertility until the age of 45 is evident from the lack of consensus among these different guidelines.

Meanwhile, although there is much documentation of the unfavorable consequences of higher maternal age on fertility, including reduced conception rates and higher risks of complications during pregnancy and childbirth [22], the impact of paternal age is less well understood [23]. To underscore the increasing relevance of this demographic shift, it is critical to recognize the corresponding rise in the utilization of IVF, reflecting changes in reproductive strategies. Over the past 30 years, there has been a significant increase in the number of IVF cycles initiated, driven by greater societal acceptance and advancements in reproductive technologies. This trend correlates with the expanding body of research emphasizing the influence of paternal factors on ART outcomes. Notably, the data from the Society of Assisted Reproductive Technology (SART) indicate that the average number of embryos transferred per cycle has been significantly reduced since the late 1990s, paralleled by a steady increase in the number of pregnancies and live births per cycle, highlighting the evolving efficiency of ART practices [24].

A systematic review indicated that paternal age did not affect ART outcomes significantly up to the age of 45, but beyond this age, there seems to be a decline in pregnancy and live birth rates. This suggests that 45 could be considered a cautionary threshold for evaluating ART outcomes [25]. Studies show that increasing paternal age is linked to diminished testicular function [26], leading to decreased sperm concentration and motility [27,28]. This decline in semen quality, particularly evident in men over 40, has been associated with reduced fecundity and higher miscarriage rates [29]. In the context of ART, these changes have significant repercussions. Research studies have shown that among couples who opt for in vitro fertilization (IVF), the chances of a couple getting pregnant are smaller in males aged over 40 [29], a trend that is similarly reflected in lower fertilization rates [30], live birth rates [31], and embryo quality scores [32]. This age-related decline in male fertility factors underlines the need for scrupulous consideration of paternal age in fertility treatments and family planning.

Furthermore, the implications of these changes extend beyond reproductive success in ART. The reduction in semen quality and fertility rates in older men can lead to increased psychological stress and anxiety related to infertility and the ART process. As such, the integral role of paternal factors in ART outcomes cannot be understated, necessitating a holistic approach that takes into account both the biological and the psychological aspects of late fatherhood [33].

Geopolitical and cultural dynamics also play a role, often leading to delayed parenthood and signifying a shift in societal norms and expectations regarding fatherhood [34]. Additionally, postponing fatherhood extends beyond mere physical implications. Studies have shown an increase in depressive symptoms among men who defer fatherhood, highlighting potential mental health consequences [35]. The use of gestational carriers has also risen, driven by increased awareness, broader access to ART services for various patient groups, and evolving surrogacy laws, all of which impact paternity trends [36].

The gradual increase in the age at which men become fathers for the first time reflects changing social and cultural norms [37]. Fatherhood significantly impacts men’s cognitive and emotional well-being, often leading to unease and uncertainty [38]. Additionally, the challenges young fathers face in balancing work and parenting further reinforce the decision to postpone fatherhood [39,40].

In essence, the use of ART among men is dictated by a complex interplay of social, cultural, and economic influences, ranging from shifts in societal standards and cultural expectations to the psychological impact of fatherhood on employment, as well as biological aspects. A comprehensive understanding of these factors is crucial in effectively managing the consequences of delayed fatherhood and optimizing the use of ART.

## 3. Aging and Its Impact on Male Reproductive Health: Anatomical, Cellular, and Molecular Perspectives

**Figure 1:**
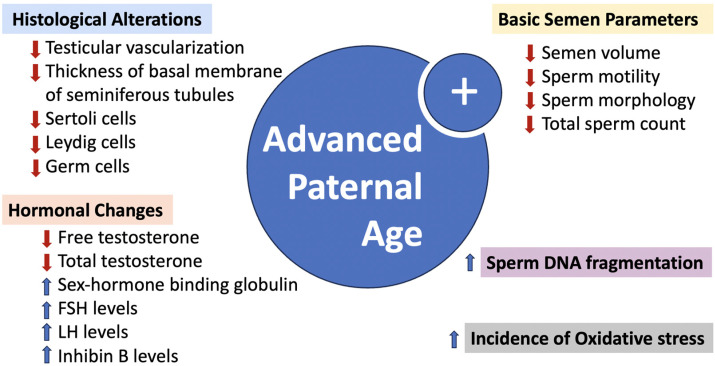
Effects of advanced paternal age on the male reproductive system.

Aging in men brings about notable anatomical changes in the reproductive system, particularly within the testes, which are critical for maintaining healthy spermatogenesis [41]. Figure 1 below illustrates the comprehensive effects of aging, including anatomical alterations, decreases in semen parameters, hormonal changes, oxidative stress, and DNA fragmentation, highlighting the multifaceted nature of age-related decline in male fertility.

As men age, there is a reduction in the number of Sertoli cells, possibly due to compromised blood flow to the testes. This decline is often accompanied by a drop in proteins that are secreted by Sertoli cells, loss of germ cells, and heightened cell apoptosis; moreover, the number of Leydig cells responsible for testosterone production also diminishes with age [42]. This leads to a disruption in hormonal balance, specifically in the levels of testosterone, luteinizing hormone (LH), and follicle-stimulating hormone (FSH), altering the hormonal milieu essential for spermatogenesis [43]. In older men, the amount of FSH increases in response to elevated inhibin B levels, a regulatory mechanism for activity in the testes [7].

These cellular changes are compounded by a general decline in the structural integrity of the testicular tissue. With age, the testes may exhibit signs of fibrosis and increased deposition of extracellular matrix, contributing to a thicker basement membrane [44]. These histological alterations can impact the overall functionality of the testes, including reduced efficiency in sperm production and a potential decrease in overall sperm quality. Additionally, the testicular vasculature undergoes changes, often leading to compromised blood flow, which can further affect the health and function of testicular cells [45].

The relationship between traditional semen quality measures (count, motility, and morphology) and age remains a topic of debate, with various studies yielding inconsistent findings [46,47,48,49,50]. To quote one example, Frattarelli et al. noted a drop in semen volume and motility in males aged over 45 [51], and Bellver et al. reported a strong negative association between father’s sperm motility and concentration [52].

Likewise, Girsh et al. and Duran et al. observed a considerable reduction in a number of sperm parameters among males over 50 years of age as compared to those under 40 [53,54]. In contrast to these results, studies by Beguería et al. and Ferreira et al. presented varying findings, with Beguería et al. noting a decrease in semen amount and motile sperm count but an unexpected increase in sperm concentration and Ferreira et al. observing no noteworthy link between sperm parameters and paternal age in normozoospermic and oligozoospermic individuals [55,56].

The inconsistent findings may be attributed to the perplexing influence of variations in ejaculatory frequency that often occur with male aging [57]. These inconsistencies may also indicate variations across research in the level of detail in the basic semen analysis. Studies have consistently demonstrated a decrease in key sperm parameters such as semen volume, sperm count, motility, morphology, and viability as paternal age advances [14,16,58,59]. Notably, a marked reduction in semen volume, which starts at 45 years old, has been reported, this being expected to diminish by 0.22 mL every 5 years (*p* < 0.001) [55]. Sperm motility has also been reported to decrease annually by 0.6% to 0.5% starting from the age of 40 (*p* < 0.001) [60].

The application of Computer-Assisted Sperm Analysis (CASA) for detailed evaluation of sperm movement characteristics consistently reveals a marked decrease in sperm motility quality as paternal age increases, a finding that has been recurrently observed across various studies [61,62,63]. Aging can impact aspects of sperm function that are not typically evaluated in standard semen analyses. Studies have shown that advanced paternal age leads to significant changes in sperm ultrastructure [64] and alterations in the sperm proteome [65,66,67]. These variations can impact the integrity of structures and proteins that play a crucial role in sperm motility and fertilization. In addition, a comprehensive examination of the sperm’s metabolome and proteome revealed that paternal age affects certain proteins involved in energy metabolism and oxidative stress [67]. This observation is significant because it aligns with other findings indicating that oxidative stress plays a crucial role in the reduction of male fertility as individuals age, as predicted by the free radical theory of aging [68].

## 4. Paternal Aging: Effects on Sperm Integrity, Epigenetics, and Reproductive Health Implications

In addition to traditional semen parameters, there has been an examination of the genetic traits of sperm as related to paternal age. With advancing age in males, there is an increased vulnerability of the germ line to oxidative damage, characterized by lipid peroxidation, a reduction in the effectiveness of DNA repair processes, and a significant augmentation in DNA fragmentation [69].

This rise in oxidative damage to the germ line could be attributable to a diminishment in the levels of antioxidant protection that these cells have accumulated over the years [70]. This oxidative-induced damage to sperm DNA predominantly takes place during the postmeiotic phase of the male gamete’s development [71,72,73,74]. Several research studies have observed a striking link between the father’s age and sperm chromatin packing, indicating a decline in the integrity of sperm chromatin with age [75]. Meanwhile, other research has reported a rise in sperm DNA fragmentation in males aged 50 years and above, correlating with lower rates of blastocyst formation, although not with the proportion of high-quality blastocysts [76]. Additionally, some studies have highlighted the fact that, beyond DNA fragmentation and oxidative stress, advanced paternal age adversely affects sperm chromatin integrity, encompassing not only the integrity of DNA strands but also the higher-order structural and epigenetic organization of chromatin. This includes modifications of histones, changes in the ratio of histones to protamines, and alterations in the spatial conformation of chromatin. These factors are crucial in determining the success of ART procedures as they influence the functional capacity of sperm to successfully fertilize an oocyte and support normal embryonic development [14,77,78]. Moreover, increased concentrations of sperm reactive oxygen species (ROS) linked to the older age of fathers may be lessened through the addition of antioxidants. The latter intervention could potentially enhance both the rates of fertilization and implantation, as well as embryo quality [79,80].

As men age, their testes are increasingly subjected to oxidative stress, which activates telomerase activity and consequently leads to an increase in sperm telomere length (STL) within the germ line. This change in STL, which is crucial for chromosome stability and cellular division, is a notable aspect of how paternal age affects sperm characteristics. Regarding male fertility, several studies have observed a clear correlation between telomere length and oxidative stress. More specifically, heightened levels of oxidative stress have been correlated with reduced sperm telomere length, while moderate oxidative stress appears to increase telomere length [81]. Longer telomeres extend the replication capacity of cell lineages before reaching the Hayflick limit and apoptosis. This increased replication might raise the risk of mutations and prolonged exposure to environmental hazards. Recent research suggests a connection between longer telomeres and increased cancer risks, including melanoma and lung adenocarcinoma [82]. Some research studies have demonstrated a positive connection between embryo quality and STL without this necessarily being associated with pregnancy rates [83]. The longer STL in older men may result from elevated testicular telomerase activity or the selective depletion of spermatogonial stem cells with shorter telomeres, a phenomenon known as ‘selfish spermatogonial selection’ [84].

Intriguingly, while paternal age does not significantly affect overall sperm aneuploidy rates, with research showing no major differences in chromosomal aberrations between older and younger men [85], among older men, observations have been made of a higher prevalence of chromosomal deletions, duplications, and balanced translocations in their sperm. This may be attributable to accumulated mutations during spermatogenesis and sperm transit [86,87]. Significantly, approximately 75% of new mutations in the human species stem from male reproductive cells and are tightly linked to paternal age [88]. These mutations are diverse and may result from replication errors and insufficient repair of DNA damage within male reproductive cells, which is exacerbated by age. It is important to note that mainly single-strand DNA breaks are repaired and less often double-stranded ones, predominantly involving the base excision repair (BER) DNA repair system. Moreover, up to 8% of sperm nuclear DNA damage can be repaired by the oocyte post-fertilization. The sperm is equipped with the OGG1 enzyme, which detects and initiates the repair of oxidative DNA damage. As a result of its action, abasic (AP) sites are created, which, if not properly repaired after fertilization, may lead to mutations [89]. DNA damage in sperm increases exponentially with paternal age [90], contributing to issues observed in ART outcomes and early pregnancy loss [91,92]. Post-fertilization, any imprecise repair of sperm DNA damage can lead to mutations affecting offspring health. This ‘post-meiotic oocyte collusion theory’ suggests a collaborative influence of both male and female germ lines in mutation formation, with older age impacting DNA integrity in spermatozoa and the DNA repair capacity of the oocyte [90,93,94,95].

Furthermore, oxidative stress in sperm due to aging particularly affects chromosome 15, which is known to be linked with various neuropathological conditions and sensitive to oxidative damage [95,96]. Such susceptibility to oxidative stress, especially within the interlinker regions of sperm chromatin, may lead to mutations associated with a range of disorders often associated with the older age of the father, emphasizing the complex interplay between oxidative stress, the father’s age, and the risk for genetic disorders in offspring [97,98,99,100].

Epigenetic modifications in older males, specifically in DNA methylation patterns and chromatin structure, are garnering significant research interest due to their potential impact on offspring phenotypes, embryo development, and embryonic transcription [33,34]. With advancing paternal age, sperm DNA methylation signatures undergo notable changes affecting gene function [16,101]. Aging male spermatozoa exhibit several DNA methylation changes, particularly in CpG regions that regulate genes associated with neurological, psychiatric, and behavioral disorders. Such disorders, with a higher incidence in children of older fathers, include spontaneous schizophrenia, bipolar disease, mood disorders, and autism [86,102]. Recent studies suggest that sperm cell epigenetic profile alterations are linked to autism-related changes in offspring, indicating a critical mechanism by which paternal age influences child health and well-being [103].

The exact molecular processes behind the latter epigenetic alterations in the male germ line remain under investigation but may be related to oxidative stress, a common aspect of male reproductive aging. Oxidative stress can alter various epigenetic components in spermatozoa, including DNA methylation, non-coding RNA species, and the amount and methylation status of histones [104,105,106]. The effect of age-related oxidative stress on the epigenetic state of men’s reproductive cells and its subsequent effects on offspring health is a burgeoning area of study that promises to significantly enhance our understanding of paternal influences on development [107].

## 5. Impact of Advanced Paternal Age on ART Outcomes

Although the adverse effects of the mother’s age on ART outcomes have been richly documented, the effect of older paternal age comprises a more complex and less understood landscape. This section delves into the nuanced impacts of paternal age on several features of ART, including fertilization rates, embryo quality, implantation, miscarriages, and live birth rates. Table 1 below summarizes the impacts of advanced paternal age on ART outcomes and potential mitigation strategies, providing a concise overview of the challenges and solutions identified in this study.

Despite conflicting results and limited data, emerging trends highlight the intricate links between paternal age and ART outcomes [53,108,109,110,111].

### 5.1. Impact of Paternal Age on Fertilization Rates

Numerous studies have examined the impact of paternal age on fertilization rates, particularly within the context of ART, revealing a pattern of decline in older male cohorts. Luna et al. noted a significant decrease in conception rates using IVF among men aged 50 and above, considering the age of egg recipients [27]. Kaarouch et al. similarly observed reduced fertilization rates in males over 40 compared to younger men. However, in cases employing intracytoplasmic sperm injection (ICSI) [108], no unfavorable impact of paternal age was reported on rates of fertilization (*p* = 0.008) [53]. Tiegs et al. additionally observed a drop in fertilization rates as paternal age increased, even after adjusting for the age of egg donors [112]. Moreover, Duran et al. [53] saw a considerable diminishment in fertilization rates that correlated with paternal age in the ICSI subgroup, in particular when a comparison was made between men aged 25–38 and those aged >50.

By contrast with the above findings, other studies present a varied perspective. Johnson et al. pointed to a trade-off between aging in males and early offspring fitness, suggesting a potential influence on fertilization [113]. Hassan & Killick argued that standard sperm evaluations might not fully capture the fertilizing capacity in the context of aging [114]. Meanwhile, Wu et al. reported no significant impact of paternal age on fertilization rates. Collectively, the latter studies point to intricate links between the father’s age and ART outcomes, with evidence indicating a decline in fertilization ability in older males, though some results showed no clear effect [115]. This underscores the need for further research to unravel the enigma concerning how advanced paternal age influences ART success.

The effects of advanced paternal age on fertilization rates using ART are complex and multifaceted, stemming from various biological mechanisms. As men age, their risk of de novo mutations of sperm increases, potentially altering its genetic integrity and impairing its fertilizing power [116,117]. Changes to genetic imprinting due to aging may alter gene expression patterns critical for early embryonic development, potentially decreasing fertilization success and leading to difficulties during fertilization [118]. Advanced paternal age has also been linked to an increased risk of genetic diseases that could reduce sperm’s effectiveness at fertilizing an egg effectively, as well as declining quality due to DNA fragmentation and chromosomal anomalies [107]. Age-related changes to sperm contribute to both decreased quantity and quality, further decreasing fertilization rates. Genetic and epigenetic alterations suggest a complex but clear pathway through which advanced paternal age influences fertilization outcomes in ART.

### 5.2. Effect of Paternal Age on Embryo Quality

There has been an ever-increasing focus in recent research on the impact of older paternal age on embryo development and quality in ART. A variety of studies have shown that as men age, there are discernible changes in embryo development rates and quality. A study by Luna et al. reported a drop in the quantity of embryos, having over seven cells on day 3, while a slower pace of blastulation with increasing paternal age [27]. In a study using an egg donation model for ICSI, Girsh et al. found a significant age difference between males in the pregnant group (average age 43.2 ± 8.1 years) and the non-pregnant group (average age 46.8 ± 7.8 years), with a lower proportion of high-quality embryos on day 3 in the non-pregnant group [54]. Frattarelli et al. reported a decline in blastocyst development in males over 55 years, noting a reduction in blastulation rates starting from age 50 [51]. Similarly, Chapuis et al. identified a decrease in blastocyst development rates in couples where the male partner was over 50 years old during IVF procedures [119].

Complementing these findings, García-Ferreyra et al. associated older paternal age with higher aneuploidy rates in embryos coming from donated oocytes, signifying a potential impact on chromosomal abnormalities and embryo quality [120]. Wu et al. provided further insights when analyzing the effect of paternal age on various aspects of embryo quality, such as fertilized oocytes and viable embryos [121]. García-Ferreyra et al. also underlined the correlation between advanced father’s age and aneuploidy rates [76].

Adding to this, a recent meta-analysis reported increased rates of blastocyst formation and a higher number of cleavage-stage embryos when the paternal age was under 50 years, again highlighting the controlled maternal age as a significant factor [122]. These findings underscore the nuanced impact of paternal age, suggesting that its negative effects may be mitigated in contexts where maternal factors are standardized.

These studies collectively highlight a potential relationship between higher paternal age and various facets of embryo quality, including aneuploidy rates, blastocyst development, and the overall quality of embryos. They suggest that advanced paternal age could well be related to impaired embryo development and a heightened risk of chromosomal abnormalities, thus impacting reproductive outcomes in ART. The need for further research to elucidate the mechanisms behind these observations and to develop strategies to enhance embryo quality in ART is evident from these diverse and insightful studies.

Advanced paternal age leads to significant genetic and epigenetic changes that adversely affect embryo development and quality. As men age, the structural integrity of sperm DNA diminishes, resulting in increased fragmentation. This fragmentation can impair early embryonic development, compromising both the quality and viability of embryos [86]. Additionally, sperm from older males are more prone to chromosomal anomalies, such as aneuploidies and Y chromosome microdeletions. These abnormalities can disrupt chromosomal segregation during embryo development, increasing the likelihood of miscarriages and diminishing embryo quality [78].

Epigenetic alterations, including changes in DNA methylation and histone configurations, also significantly influence development by altering gene expression in sperm. These modifications can be inherited by embryos, disrupting normal developmental processes and potentially leading to delays or abnormalities [7]. Moreover, these genetic defects and epigenetic changes contribute to what is termed ‘sperm genome decay’, where the overall genomic integrity of sperm is compromised. Research has shown that this decay negatively affects the sperm’s fertilization capability, thereby reducing the quality of embryos and the success rates of ART outcomes [108].

Considering these factors is crucial when assessing embryo quality in ART settings. Implementing genetic screening for egg donor cycles using sperm from older men is recommended to identify and mitigate the risks associated with these genomic and epigenetic changes [123].

### 5.3. Effect of Paternal Age on Implantation

The influence of paternal age on implantation rates in ART has been the subject of several significant studies, each contributing unique insights into this complex relationship. Khandwala et al. conducted a comprehensive cohort study which, while not directly focused on implantation rates, revealed that higher paternal age is related to higher rates of premature birth, low birth weight, and lower Apgar scores [124]. These findings, though indirect, provide valuable context for an enhanced understanding of the broader implications of paternal age on pregnancy outcomes.

Luna et al. examined ART outcomes in ovum recipients, uncovering a possible impact of paternal age on pregnancy outcomes and blastocyst formation rates [27]. Though their research did not explicitly target implantation rates, it pointed to a potential link between paternal age and overall success in ART procedures. In contrast to the above results, Wu et al. explored the reproductive outcomes of in vitro fertilization while reporting a range of differing findings and proposing that paternal age does not appreciably affect implantation rates [115]. This highlights the complexities and variabilities involved in determining the specific effects of paternal age on implantation.

Further adding to the conversation, Meijerink et al. observed that embryo implantation rates tend to decrease as paternal age increases [125]. However, this study also noted inconsistencies regarding overall pregnancy outcomes and did not account for the age of the recipient, indicating the need for a more nuanced understanding of these dynamics [125]. Setti et al. offered a more comprehensive view, reporting that paternal age adversely affects several key factors in ART, including fertilization rates, the quality of embryos on day 3, blastocyst formation, and implantation rates [126]. The latter study additionally identified correlations between advanced paternal age and lower rates of pregnancy and live births.

Collectively, the aforementioned studies point to a nuanced association between advanced paternal age and implantation rates in ART procedures. While some research indicates a potential detrimental effect of increased paternal age on implantation and subsequent pregnancy outcomes, other findings present a more complex and less definitive picture. This diversity in research outcomes underscores the necessity for further studies in this area in order to more accurately decipher the part played by paternal age in implantation success and, overall, ART outcomes. In summary, although evidence exists indicating a correlation between advanced paternal age and implantation rates in ART, the exact nature and scope of this impact still remain areas of active investigation and debate.

The impact of advanced paternal age on embryo implantation in ART involves various factors that have been investigated in several studies. It has been observed that the transfer of euploid embryos resulted in an implantation rate of approximately 60%, while almost 40% of embryos remained unaccounted for, indicating a potential impact of paternal age on implantation rates [127]. Ferreira et al. documented that paternal age could adversely impact implantation and pregnancy rates, particularly in cases of oligozoospermia [56]. Furthermore, a decrease was reported in implantation and pregnancy rates with paternal age in specific maternal age groups, suggesting a potential influence of paternal age on implantation outcomes [121].

Advanced paternal age has been associated with an increase in sperm DNA fragmentation, contributing to blastocyst formation failure and poor clinical outcomes, which could impede embryo implantation [76]. This age-related genetic degradation can compromise embryo integrity by interfering with the early stages of embryo development, leading to lower implantation rates [108]. Furthermore, increased ROS concentrations in sperm associated with older paternal ages may damage sperm and embryo DNA. However, interventions like the addition of idebenone have been shown to lower ROS levels, thereby potentially enhancing embryo quality and improving implantation rates after IVF [79].

Moreover, embryos from older fathers often exhibit decreased quality, evident from higher miscarriage rates and reduced implantation and live birth rates, likely due to increased genetic anomalies and epigenetic modifications prevalent in older males [78]. Despite these findings, Dain et al. report no clear correlation between advanced paternal age and ART outcomes, underscoring the variability and complexity inherent to these procedures and emphasizing the need for further research to fully understand these dynamics [35].

### 5.4. Effect of Paternal Age on Miscarriages

The extensive discussion surrounding the risks of miscarriage and other negative reproductive outcomes in older men who undergo ART is complex and nuanced. Although several studies tend to suggest that there is no major link between paternal age and the rates of miscarriage or live births, the situation changes slightly when men reach the age of 50 or above [51,110,115,128,129]. After reaching a certain age, a noticeable pattern appears wherein the number of live births tends to decline while the likelihood of miscarriage increases. The risk of miscarriage notably rises, especially beyond the age of 50 [130], and continues to climb even after men reach 40 years old [131]. In addition, offspring of males above 45 are more likely to experience premature birth, with a reported 14% increase in risk [124].

However, a recent meta-analysis has shown that paternal age under 50 years significantly reduces the miscarriage rate in donor oocyte cycles, where maternal age is controlled [122]. This introduces a critical perspective into the ongoing debate about the effects of paternal age, particularly under controlled maternal age conditions.

Nevertheless, there is, at present, no consensus in the literature, and the interpretation of these results is susceptible to various biases. Maternal age often serves as a confounding variable that may influence results [52,55,109,112,132,133,134,135]. In order to elucidate this issue, researchers have used IVF data obtained from oocyte donation programs as a means of partly circumventing the danger associated with maternal age. However, the age of the pregnant woman is still a risk factor for giving birth prematurely [136]. Besides age, several variables such as paternal smoking, the type/cause of infertility, the number of previous efforts to achieve ART, maternal smoking habits, and alcohol consumption may significantly impact ART results, hence contributing to the intricacy of risk assessment [129,131].

While the results vary, the overall pattern indicates that a careful approach should be used when contemplating ART for older males. Although 12 studies did not discover a substantial connection between the age of fathers and either miscarriage or live births, other research has shown a decline in the percentage of successful live births and an increase in spontaneous miscarriages as men age. These results emphasize the need for meticulous preimplantation genetic screening as well as the necessity to take into account all possible factors that might affect the evaluation of risks related to advanced paternal age in assisted reproductive technology procedures.

Research has consistently demonstrated that paternal age plays a significant role in miscarriage rates. Men, as they age, experience increased sperm DNA fragmentation rates that compromise embryo development and raise miscarriage risk [108]. Furthermore, studies indicate these age-related changes may lead to an accumulation of chromosomal abnormalities within sperm that, when fertilizing an egg, may result in embryos with genetic defects predisposing them to miscarriage [86,137].

Additionally, advanced paternal age has been found to negatively impact sperm quality overall, manifesting as increased abnormal sperm cells and reduced motility; such changes reduce both successful conception rates as well as increasing risks for poor embryonic outcomes once conception does take place [138,139]. Such findings highlight the intimate link between paternal age and increased miscarriage rates, thus emphasizing the necessity for comprehensive genetic screening, as well as carefully considering all paternal factors when assessing ART risks and outcomes.

### 5.5. Effect of Advanced Paternal Age and Perinatal Risks

The effect of advanced paternal age on certain birth complications, namely, the risk of premature birth, low birth weight, and stillbirth, constitute a crucial area of study in the context of ART procedures. Indeed, a wealth of research has identified a clear association between advanced father’s age and a number of unfavorable perinatal outcomes.

Risk of Premature Birth: Advanced paternal age has been associated with a higher risk of premature birth. Studies suggest that fathers aged 40 to 45 years and above may contribute to a higher incidence of preterm births, with infants also facing increased risks of low Apgar scores, a measure of the physical condition of a newborn [124]. A population-based cohort analysis found that 13.2% of premature births were associated with older fathers, indicating a notable correlation between advanced paternal age and the risk for preterm delivery in ART outcomes [124]. Additionally, the same study revealed that higher paternal age is associated with an elevated risk of preterm delivery, gestational diabetes, and neonatal convulsions [124]. After accounting for many confounders, it was shown that the odds ratios of birth abnormalities such as cleft lip, diaphragmatic hernia, right ventricular outflow tract blockage, and pulmonary stenosis increased dramatically with each additional year of paternal age [140].

However, some studies indicate that the father’s older age may not be independently linked to any risk of very early preterm delivery [141]. Moreover, another study suggests that in ART-treated and subfertile couples, no association can be identified between older paternal age and a higher risk of prematurity, low birth weight, or small for gestational age, highlighting the variability of findings and the need for further research [133]. This underscores the complexity of assessing risks and the importance of considering individual circumstances and a range of contributing factors when evaluating the potential impacts of advanced paternal age on birth outcomes.

Risk of Low Birth Weight: Advanced paternal age has been linked to a higher risk of low birth weight in neonates, indicating that there is a greater possibility of infants born to fathers of an older age weighing less than what is considered normal for their gestational age at birth. This association holds even when accounting for the age of the mother, a traditionally recognized factor in birth weight [142]. Advanced paternal age is also associated with an increased risk of low birth weight in ART outcomes. The study by Khandwala et al. further reported gestational diabetes in 18.2% of births associated with older fathers, which can be a contributing factor to low birth weight [124]. This underlines the importance of taking paternal age into account in the context of ART treatments and potential birth outcomes, highlighting the multifaceted implications of older paternal age on infant health.

Stillbirth Concerns: Advanced paternal age has been associated with a higher rate of stillbirths, particularly in the case of fathers aged above 40. This suggests a link between the father’s age and the viability of the pregnancy, with older paternal age contributing to increased risk during the gestational period [143,144]. It has been observed that fathers of ages 40–45 have a 24% higher risk of their partner suffering stillbirth; this, however, is accompanied by a lower risk of small for gestational age (SGA) infants [145]. The association between older fathers and a higher rate of stillbirths is a significant concern in ART outcomes [145]. This underscores the need for careful consideration and potentially additional monitoring in pregnancies via ART involving older fathers, emphasizing the delicate balance and the complex implications of advanced paternal age on pregnancy outcomes.

Of note, the above adverse outcomes may be influenced by various confounding factors such as maternal age, lifestyle factors (smoking, alcohol consumption), and the type of infertility being treated. An association has also been noted between advanced paternal age and higher risk of gestational diabetes among mothers and infants, complicating the perinatal period [146]. While important associations have been noted between advanced father’s age and a number of adverse birth outcomes in the framework of ART, it is essential to consider that these outcomes may additionally stem from other factors, such as the specific ART methods used and individual health conditions.

While the research indicates a clear trend towards increased risks with advancing paternal age, the extent and nature of these risks can vary greatly, with individual outcomes depending on a multitude of factors. The findings also highlight the variability and complexity of the impacts of advanced paternal age on ART outcomes, suggesting that a personalized approach might be necessary for the management of pregnancies involving older fathers. This evidence forms a crucial component of the current article discussing the effects of advanced paternal age on ART outcomes.

In conclusion, it is clear that advanced paternal age has, in many cases, been associated with a higher risk of complications in ART, including premature birth, low birth weight, and stillbirth. These findings should be integrated into clinical practice to optimize the care and counseling provided to couples undergoing ART, especially where the paternal factor is a concern.

Research into the biological mechanisms underlying the association between advanced paternal age and higher perinatal risks has provided us with important insight into how genetic and epigenetic changes within sperm may influence these outcomes. As men age, their sperm becomes subject to genetic mutations and epigenetic modifications that may significantly compromise the health and development of an embryo. Studies have documented an accumulation of DNA mutations in sperm from older fathers that can lead to developmental abnormalities and compromise pregnancies [7,8]. Epigenetic changes often exacerbate genetic modifications, as changes to DNA methylation patterns can influence gene expression within an embryo and impact its survival and development [86].

Age also diminishes the quality of semen, leading to decreased motility and increasing incidences of abnormal sperm. These changes reduce the probability of successful fertilization while simultaneously increasing miscarriages and low birthweight births [108]. Furthermore, advanced paternal age could serve as a proxy for other risk factors that accumulate with age, such as health conditions or environmental exposures that impact offspring’s health and further complicate perinatal risks associated with older fathers [147].

Understanding these mechanisms is vital in order to effectively assess risks in ART treatments, and it stresses the significance of paternal age as a significant factor in prenatal care and counseling. With this knowledge comes an ability to create targeted interventions that may reduce risks and improve perinatal outcomes.

### 5.6. Effect of Paternal Age on Live Birth Rate

Campos et al. discovered a detrimental impact of increased paternal age on clinical pregnancy within the context of oocyte donation [111]. However, the authors noted that maternal age might potentially be a confounding factor. Specifically, the negative association between pregnancy and age became insignificant when one of the parents was below the age of 39. Wu et al. conducted a research study that found a decline in clinical pregnancy rates among males over the age of 36 compared to those under the age of 32 [115]. The latter was demonstrated among couples of whom the mother was aged 30–34 years. In a similar manner, Ferreira et al. assessed the results of ICSI based on the age of the father in men with normal sperm count and men with low sperm count [56]. Upon adjusting for mother’s age, number of oocytes extracted, sperm concentration, and rate of fertilization, the authors observed a drop in implantation and pregnancy rates, particularly among older fathers with oligozoospermia. The researchers reached the conclusion that the likelihood of successful pregnancy decreases by 5% for every additional year of paternal age in individuals with oligozoospermia [56]. Various studies have established a connection between greater paternal age and a longer duration needed for conception after the age of 34 [12], as well as a reduced success rate in ARTs [14].

In a study conducted by Park et al. on males with azoospermia, it was shown that couples with male partners beyond the age of 46 had lower clinical pregnancy rates, regardless of the etiology of azoospermia [148]. Frattarelli et al. observed a direct correlation between higher rates of pregnancy loss and older paternal age [51]. The study revealed a notable disparity in the rates of pregnancy loss between males under the age of 50 (41.5%) and those above the age of 50 (24.4%; relative risk 0.61, 95% confidence interval: 0.45–0.84; *p* < 0.01) [51]. Additional research indicated a tendency towards higher rates of pregnancy loss among dads of advanced age; however, the findings did not achieve statistical significance [119].

A number of studies have investigated the effect of paternal age on the frequency of successful live births [51,128]. McPherson et al. observed a decline in LBR in couples with advanced maternal age (>35) and paternal age (>40) [128]. The authors postulated that the detrimental impact of advancing age on SDF (sperm DNA fragmentation), the oocyte’s cytoplasmic DNA repair systems, and endometrial receptivity could account for the suboptimal reproductive results observed in couples of older age. Nevertheless, the authors of the above study cautioned that the damaging effect of paternal age on ART results is, in fact, a good deal less significant than the influence of the mother’s age [128]. Subsequent to artificial insemination, the pregnancy rate declined by half among males aged 35 and over [149], while following IVF, the pregnancy rate was considerably altered among men aged 50 and over [30].

In contrast, other groups did not demonstrate any influence of paternal age on ART outcomes. In a research study conducted by Bellver et al., it was shown that there were no statistically significant variations in pregnancy rates and miscarriage rates across various paternal age categories [52]. Within the subset of egg donors, the researchers observed a rise in embryo fragmentation linked to paternal age. However, the association was minimal, casting doubt on its clinical relevance [52]. In a separate research study including 278 couples, Nijs et al. noted no discernible variations in fertilization rates, pregnancy rates, and live birth rates when the age of the mother was taken into account [75]. However, this research work excluded males with severe oligoasthenoteratozoospermia (OAT), men using testicular sperm for ICSI, and instances that included preimplantation genetic testing.

It is of note that most studies that indicated no impact of paternal age on ART results had limited sample sizes and a smaller number of males over the age of 40 [52,150,151]. However, other research work included large groups of participants and considered such factors as the age of the mother and the quantity of embryos transplanted in each reproductive cycle. Meijerink et al. carried out a study in which they assessed the effect of paternal age on the first attempt at ART in 7051 couples [125]. The authors additionally evaluated the influence of paternal age based on the origin of sperm used for ICSI, specifically, ejaculated sperm and sperm obtained by procedures such as percutaneous epididymal sperm aspiration (PESA), microsurgical epididymal sperm aspiration (MESA), and testicular sperm extraction (TESE). The latter wide-ranging research study reported no association between paternal age and continued pregnancy rates achieved via ART. Furthermore, the source of sperm did not have any impact on the outcomes of pregnancy. In contrast, Whitcomb et al. noted no negative effect of paternal age in a study comprising 1083 couples who used an oocyte donor [152]. This finding remained consistent even after accounting for the female partner’s age.

A study by Begueria et al., which examined an egg donation model using ICSI as the only fertilization method, reported that male age did not have any impact on pregnancy outcomes [55]. The latter method includes the rates of biochemical pregnancy (RR: 1.0; 95% CI: 0.96–1.05), miscarriage (RR: 1.06; 95% CI: 0.94–1.03), ongoing pregnancy (RR: 0.98; 95% CI: 0.94–1.033), and live birth (RR: 0.98; 95% CI: 0.94–1.03) [55]. Ghuman et al., who carried out a study on the effect of advanced paternal age (i.e., 40–45 years of age) in a sperm donation method, reported analogous results. After considering female age, treatment technique, and the impact of prior treatment cycles, no correlation was noted between the father’s age and miscarriage or LBR. Despite these findings, the authors warned that it may not be feasible to apply their conclusions to a wider population since the sperm used in the study came from a specific group of donors, even though most men seeking assisted reproductive technology had abnormal sperm characteristics [109].

In summary, while there are minimal data indicating a potential detrimental effect of greater paternal age on ART outcomes, the possible association between these two factors remains uncertain. Among all of the research studies, it is evident that maternal age is the primary determinant of pregnancy success in IVF and ICSI.

Advanced paternal age profoundly impacts reproductive outcomes, primarily due to increased SDF. As men age, there is an increase in DNA mutations and chromosomal aneuploidies, which are linked to reduced fertilization rates, higher miscarriage rates, and an increase in congenital anomalies. Such genetic risks significantly compromise embryo quality, reducing implantation rates and live birth rates from IVF/ICSI treatments, especially notable in fathers over 40 years of age [7,153].

Moreover, the combined effects of paternal and maternal ages intensify these risks, with advanced age in both parents exacerbating the decline in live birth rates. Interestingly, the negative impacts of older paternal age on live birth outcomes are somewhat mitigated when the female partner is under 35, suggesting that younger maternal age can buffer the adverse effects of aged sperm [128]. Furthermore, advanced paternal age is linked to a higher risk of adverse birth outcomes such as low birth weight, preterm birth, and stillbirth, which indirectly affect the live birth rate by compromising pregnancy viability [154,155].

The negative impacts of advanced paternal age on live birth rates arise from a complex interaction among decreased sperm quality, genetic risks, and combined maternal age effects. These findings underscore the necessity of reproductive counseling and ART protocols that take paternal age into account to optimize care and increase the chances of successful pregnancy outcomes. Further research must be conducted in order to fully comprehend these dynamics and tailor treatment plans appropriately.

## 6. Strategies for Deferred Fatherhood and Future Research Directions

ART has revolutionized the treatment of infertility. However, the influence of advanced paternal age presents unique challenges that impact the success and outcomes of ART procedures. Understanding these challenges is crucial for the development of strategies to mitigate risks and improve outcomes. Meanwhile, the exact connection between paternal age and ART results is highly complex and, thus, at present, remains beyond our understanding. While a considerable amount of research often suggests a detrimental impact, particularly for men aged 40 or above [110,128], pinpointing a specific age threshold where risks significantly increase remains challenging [111,115]. This ambiguity arises from the diverse methodologies and demographics employed in studies, along with a diversity of outcome measures, making qualitative comparisons and meta-analyses difficult. Guidelines from the Society of Obstetricians and Gynecologists of Canada (SOGC) and the American College of Obstetricians and Gynecologists (ACOG) suggest considering men aged 40 and above as “older fathers” in ART contexts [156,157] and recommend counseling regarding the risks of postponing parenthood, which entails probable diminishing success in ART procedures, as well as a number of health challenges for the offspring.

Despite the expanding corpus of research on ART in older males, numerous questions remain unresolved. At present, a definitive counseling guideline expressly designed for older men seeking reproductive assistance is notably absent. In practice, counseling is predicated on the clinical condition of the patient, the possible existence of comorbidities, and his social and cultural background. This approach underscores the necessity for a nuanced and individualized framework to address the unique needs and circumstances of this demographic.

Oral antioxidants have been extensively used, though with varied outcomes, to mitigate the adverse effects of oxidative stress on sperm in men with infertility [158]. This approach might also be considered for older males, whose sperm are more susceptible to oxidative harm. Similarly, in the broader community of men with infertility, there is as yet no knowledge concerning which could be the most efficacious combination(s) of antioxidants nor what their dose and duration should be. While antioxidant supplements could be efficacious for the enhancement of male productive health in older-aged men, it is crucial to be aware of ‘the antioxidant paradox’. This term, introduced by Halliwell et al., highlights the potential downsides to the excessive use of antioxidants [159]. Overexposure to these reducing agents can lead to reductive stress, a condition similar to oxidative stress, which might impair the fertilizing potential of the spermatozoa in a manner akin to oxidative stress itself [160].

In counseling prospective fathers, it is imperative to engage in a comprehensive discourse regarding the merits and demerits of deferring paternity. Young men contemplating postponed fatherhood might consider sperm cryopreservation as a strategic intervention to ameliorate the age-related decline in fertility. Nonetheless, the decision to opt for elective sperm freezing necessitates a careful evaluation of several pivotal factors, particularly as the thawed sperm will require utilization in conjunction with assisted reproductive techniques [161].

While the utilization of semen from a younger patient may theoretically diminish the incidence of such conditions as Down syndrome, schizophrenia, and autism spectrum disorders, the potential compromise to sperm quality and DNA integrity due to the freeze–thaw process warrants serious consideration [162]. Moreover, beyond these biological risks, it is critical to meticulously address and evaluate the associated ethical, legal, and financial implications when counseling potential fathers about sperm cryopreservation [162].

While there are established protocols for sperm storage among patients with conditions such as nonobstructive azoospermia (NOA) or those undergoing chemotherapy, the absence of explicit guidelines for sperm freezing solely for the purpose of delayed fatherhood presents a challenge [163]. Consequently, it is vital to conduct personalized discussions with each patient, thoroughly weighing the potential benefits against the inherent risks and uncertainties. This tailored approach ensures informed decision-making, aligning with each patient’s unique circumstances and aspirations [163].

When providing counseling to older males who are about to undergo ART, it is important to address the topic of preimplantation genetic testing (PGT). Preimplantation genetic testing for aneuploidy screening (PGT-a) is often used to identify abnormalities in embryos and choose healthy embryos for transfer to the uterus. This technique is especially beneficial for women of advanced age and those who have had repeated pregnancy loss. The latter approach is also applied to conduct tests for particular genetic diseases, such as monogenic and single gene abnormalities (PGT-m), as well as chromosomal structural rearrangements (PGT-Sr). The use of PGT may result in an 18% improvement in implantation rates for older women as compared to using unscreened ICSI embryos [164,165].

Furthermore, it is equally crucial to provide counseling to infertile patients, particularly those who are aging, on the potential detrimental effects of smoking, exposure to gonadotoxins, and alcohol usage since all of these factors may significantly affect fertility [166]. While couples undergoing ART frequently overlook the need for detection and treatment of underlying disorders linked to infertility, it is crucial to prioritize these features in the care of males seeking fertility, regardless of their age group. This approach has the potential to enhance the results of ART procedures [167].

The adoption of antioxidant supplementation, the pursuit of a health-conscious lifestyle, the management of underlying conditions associated with infertility, and the preservation of sperm through cryobanking constitute viable strategies for younger individuals contemplating deferred parenthood. Older persons who are considering ART, particularly those beyond the age of 50, should contemplate the incorporation of PGT-A as a prudent aid to mitigate potential genetic risks. This multifaceted approach represents a proactive stance towards reproductive planning, aligning with contemporary advancements in reproductive health and technology.

Future research directions in the setting of advanced paternal age and ART are crucial to deepening our understanding and improving outcomes. One critical area demanding further exploration is the epigenetic mechanisms that accompany advancing paternal age. In-depth research is needed to elucidate how changes in DNA methylation and histone modifications, as well as the influence of non-coding RNAs, contribute to paternal age-related reproductive outcomes. Gaining greater insight into these epigenetic alterations is essential in order to better comprehend their potential impact on ART outcomes and offspring health. Investigations into these mechanisms promise to reveal significant findings regarding potential therapeutic targets and interventions that can profoundly influence the field [107].

Another vital future direction involves the implementation of longitudinal studies, which are essential to monitor and, thereby, increase our knowledge about the long-term health trajectories of offspring conceived through ART from older fathers. By tracking these individuals over extended periods, researchers can gather invaluable data on the prevalence and nature of genetic, neurodevelopmental, and psychiatric disorders. Such long-term follow-up studies are crucial for a comprehensive understanding of the multifaceted impact that advanced paternal age has on the health and well-being of progeny [168].

Also, with regard to crucial future directions, employing sophisticated genetic and epigenetic profiling techniques enabling assessment of the impact of advanced paternal age on sperm quality, embryo development, and offspring health constitutes yet another vital area of research. Utilizing these methodologies can provide critical data for identification of biomarkers and predictive indicators essential for ART success and offspring health [169].

Simultaneously, the design and execution of interventional studies are crucial to evaluate the effectiveness of lifestyle and health interventions in mitigating the risks associated with advanced paternal age in ART. Such studies are invaluable for developing evidence-based strategies aimed at optimizing reproductive outcomes and reducing the incidence of genetic and neurodevelopmental disorders in offspring [170].

In summary, effectively addressing the challenges posed by advanced paternal age in ART requires a comprehensive and integrative approach involving clinical interventions, advancements in research, and multidisciplinary collaboration. By focusing on these research directions and potential solutions, the field of reproductive medicine is well positioned to enhance ART outcomes and improve the health and well-being of offspring conceived through ART with older fathers.

## 7. Conclusions

In conclusion, today’s growing trend towards advanced paternal age poses challenges in ART, involving declining sperm quality, genetic risks, and psychological impacts. Men aged 40 or older face increased genetic risks and sperm quality decline, though the impact of these on ART results is smaller than those arising from maternal age. Meanwhile, the offspring of older fathers are exposed to a greater risk of developing a number of health issues. In view of all the latter considerations, reproductive medicine must adapt. Strategies for men considering delayed fatherhood include early disease detection, sperm freezing, and antioxidants. Preimplantation genetic testing is an option for men over 50 using ART. A wide-ranging approach is certainly required to plot a course through the numerous complexities pertaining to advanced paternal age in order to guarantee optimal outcomes both for couples and for their children in the context of ART.

## Figures and Tables

**Table 1:** Impact and mitigation of paternal aging on ART outcomes.

Aspects of Paternal Aging	Impact on ART Outcomes	Mitigation Strategies
Semen Quality	- Decline in sperm concentration and motility, affecting fertilization rates and embryo development. Increased sperm DNA fragmentation, affecting fertilization and embryo viability.	- Lifestyle changes (e.g., diet, exercise) - Sperm cryopreservation
Genetic Risks	- Increased DNA fragmentation, leading to higher miscarriage rates and potential chromosomal abnormalities in offspring. Increased risk of gene mutations.	- Preimplantation genetic testing - Genetic counseling
Epigenetic Shifts	- Changes in sperm DNA methylation patterns, impacting embryo development and risks of neurological disorders in offspring.	- Ongoing research into epigenetic interventions and their effects
Psychological Aspects	- Increased stress and anxiety related to infertility and the ART process.	- Psychological support and counseling for prospective fathers
Impact on Embryo and Birth Outcomes	- Lower blastocyst development rates and increased risk of adverse birth outcomes (e.g., low birth weight, preterm birth).	- Long-term follow-up studies of offspring health - Genetic screening and counseling
Long-Term Offspring Health	- Higher risk of genetic and neurodevelopmental disorders in children of older fathers.	- Long-term follow-up studies of offspring health. - Genetic screening and counseling

## Data Availability

Not applicable.
